# Innovative technology and established partnerships—a recipe for rapid adaptability under emerging pandemic conditions

**DOI:** 10.14745/ccdr.v49i05a04

**Published:** 2023-05-01

**Authors:** Shamir Mukhi, Melanie Laffin-Thibodeau, Tim Beattie

**Affiliations:** 1 Canadian Network for Public Health Intelligence, National Microbiology Laboratory, Public Health Agency of Canada; 2 Canadian Paediatric Surveillance Program

**Keywords:** data collection, public health, infectious disease, informatics, surveillance, response

## Abstract

**Background:**

Aided by a collaborative partnership dating back to 2011, the Canadian Network for Public Health Intelligence (CNPHI) and the Canadian Paediatric Surveillance Program (CPSP) quickly undertook substantial enhancements to the CPSP’s data collection instruments on the CNPHI platform to characterize the impacts of the coronavirus disease 2019 (COVID-19) on children and youth in Canada. Faced with an emerging public health threat with impacts yet unknown, the objective of the intervention was to rapidly complete enhancements to existing data collection and analytical tools to enable the CPSP’s ability to characterize the impacts of COVID-19 in Canadian children and youth.

**Intervention:**

Reporting frequency from CPSP’s network of paediatric practitioners was increased from monthly to weekly, and the flexibility of detailed case data collection was substantially enhanced using complex survey instruments, interactively designed using CNPHI’s Web Data technology. To ensure their data collection proceeded along all required lines of surveillance, CPSP’s data collection tools were enhanced to collect demographic, epidemiological, microbiological and clinical data including comorbidities of cases identified.

**Outcomes:**

Less than a month after the World Health Organization declared the COVID-19 pandemic, CPSP was able to start collecting detailed weekly case data on emerging cases of COVID-19 among Canadian children and youth. By May 2020, CPSP was able to launch a detailed study, supporting research into potential risk factors for severe COVID-19-related illness in children and youth.

**Conclusion:**

In response to a novel public health threat, CNPHI and CPSP were able to implement rapid adaptations and enhancements to existing data collection instruments while fortifying their preparedness to do the same in the future, when needed. With innovative and agile technologies at the ready, this experience helps to emphasize the importance of established collaborative partnerships across public health disciplines as a factor contributing to preparedness and agility to respond to the unforeseen. Canadian Network for Public Health Intelligence’s Web Data technology showed agile adaptability and a capacity for complex and detailed data collection, supporting timely surveillance and response.

## Introduction

Emerging and re-emerging infectious disease threats continue to test our preparedness to respond in a timely and effective manner to protect public health. Researchers and public health professionals require adaptable tools that yield intelligence to advance their understanding of known threats while providing the agility to pivot in response to the unforeseen. Experiences over the past two decades, such as the emergence of severe acute respiratory syndrome (SARS) in 2003 or pandemic H1N1 influenza in 2009, have prompted an increased focus on defining the elements that contribute to enhanced preparedness. One key element of preparedness is the establishment and fostering of ongoing collaborative partnerships across public health disciplines ((1)).

One such partnership exists between the Canadian Network for Public Health Intelligence (CNPHI) and the Canadian Paediatric Surveillance Program (CPSP). Since 2011, CNPHI and CPSP have worked together to establish and enhance data collection and analysis through a secure and easy-to-use environment that started with the design, development and launch of the e-CPSP system on CNPHI. Data collection enhancements made at that time resulted in the modernization and improved timeliness of CPSP’s data collection activities by enabling the transition from a paper-based system to an electronic system for the collection of data on rare conditions and diseases from paediatric practitioners across Canada ((2)). Although a small proportion of practitioners preferred to keep using the paper-based means of reporting, these enhancements resulted in increased agility and adaptability to support the rapid collection of surveillance information on emerging issues such as Zika virus, for example ((3)).

This article discusses agile adaptations made to CPSP’s data collection tools on the CNPHI platform in early 2020 in response to the arrival of the coronavirus disease 2019 (COVID-19), enabling enhanced surveillance on the impacts of COVID-19 on children and youth across Canada. As a novel public health threat, very little was known about the epidemiology of COVID-19, particularly its impacts on children and youth. Within one month of COVID-19 being declared a pandemic by the World Health Organization ((4)), the level of detail of CPSP’s data collection was substantially increased and data collection frequency was increased from monthly to weekly. Subsequently, when healthcare providers were starting to see patients with a new condition called paediatric inflammatory multisystem syndrome/multisystem inflammatory syndrome in children (PIMS/MIS-C), the flexibility of CPSP’s data collection tools allowed the rapid adaptability needed to collect detailed information on this novel syndrome. Both the flexibility and volume of data collection were substantially increased using CNPHI’s innovative Web Data technologies to support the creation of lengthy and complex survey instruments, in English and French, distributed via the e-CPSP system on CNPHI. Web Data enabled the data collection and extraction and the analysis of information collected on a weekly cycle. Flexibility also allowed for participants to report on cases seen in prior weeks that had not yet been reported. These rapid adaptations enabled CPSP to launch three studies in one to characterize risk factors for severe illness within hospitalized cases of acute COVID-19 in children and youth, non-hospitalized cases with acute COVID-19 and chronic comorbid conditions, as well as PIMS/MIS-C, temporally associated with COVID-19. Such adaptations have enhanced the preparedness and ability of CPSP to respond quickly to emerging health concerns both presently and in the future, including subsequent waves of COVID-19 ((5)).

## The Canadian Paediatric Surveillance Program

Established in 1996 as a joint program between the Public Health Agency of Canada and the Canadian Paediatric Society, CPSP actively collects data from approximately 2,800 paediatricians and paediatric subspecialists across Canada, representing a paediatric population of over seven million Canadian children and youth. This allows CPSP to play an important role in supporting and coordinating national public health surveillance, research as well as raising awareness of childhood disorders that highly impact disability, morbidity and economic costs to society, despite their low frequency.

Importantly, this also allows CPSP to participate as an International Paediatric Surveillance Unit, engaging in international knowledge exchange across four continents through the International Network of Paediatric Surveillance Units. International collaboration is essential in the study of rare and ultra-rare conditions, and is key to understanding and tracking novel public health threats, as was demonstrated when congenital Zika syndrome emerged, and most recently during the COVID-19 pandemic.

Historically, some important examples of CPSP’s contributions include the following:

• Capturing serious adverse events related to recreational cannabis use in children and youth, following the legalization of cannabis in Canada in late 2018. The study revealed that significant harm is caused by unintentional exposures in young children through the ingestion of edibles. These findings highlight the urgent need to keep these products out of the hands of our youngest citizens and informed the Canadian Paediatric Society’s response to the legislative review of the Cannabis Act ((6)).

• Collecting data to demonstrate that although seat belts are proven to save lives, if worn incorrectly, they can lead to seat-belt syndrome and cause significant injuries, including permanent paralysis ((7)).

• Capturing data on Vitamin D rickets—a condition that, although entirely preventable, continues to be a global health problem among children, even in developed countries such as Canada ((8)).

• Capturing data that led to the creation of national clinical guidelines for paediatricians and other child health providers on the management of severe hyperbilirubinemia, stimulating practice change that ultimately improved outcomes for children and youth across Canada ((9)).

## The Canadian Network for Public Health Intelligence

Established in 2004 following lessons learned after the SARS pandemic, CNPHI is a secure, web-based scientific public health informatics and bio-surveillance platform currently serving a large number of users from a diverse array of public health disciplines within federal, provincial and territorial agencies across Canada. A wide array of specialized applications and tools support surveillance and analytical requirements, data exchange and research, alerting and intelligence generation. In addition, CNPHI Collaboration Centre technologies support coordination, collaboration and knowledge exchange among various national groups in support of decision-making, research and program implementation. Canadian Network for Public Health Intelligence’s Web Data technology is available within the suite of Collaboration Centre tools on the CNPHI platform and has proven to be an agile and flexible technology supporting rapid data collection needs. It offers an intuitive interface that allows public health users to create data fields of various types to support data collection through surveys, questionnaires or the creation of *ad hoc* databases to meet unique public health program needs. Web Data can also be applied to address more complex data collection requirements, with the support of the CNPHI team.

As a core tenet of its organizational philosophy, CNPHI, within the National Microbiology Laboratory Branch at the Public Health Agency of Canada, recognizes the fundamental importance of the collaborative partnerships formed with public health professionals across a wide array of programs and disciplines. All applications and related enhancements are developed in close collaboration with public health program experts, ensuring that the public health informatics solutions developed meet their evolving needs. Partnerships are sustained and nurtured over time, allowing for an ongoing understanding of a program’s vision. In turn, this detailed familiarity directly supports preparedness and the readiness to adapt to changing needs related to emerging or re-emerging public health threats.

## Adaptability of Web-Data technologies in various settings

Canadian Network for Public Health Intelligence’s Web Data has continued to evolve, benefitting from the advancement of technologies and experience gained in supporting public health stakeholders across various disciplines for a number of years and under various conditions. Web Data is a technology available on the CNPHI platform, designed to alleviate challenges related to agile data collection during outbreaks. It provides a mechanism for non-technical users to rapidly deploy a secure, web-based system for managing data and undertaking subsequent analyses and reporting. Developed in 2008 by CNPHI, Web Data was first put to the test, and its effectiveness evaluated, in partnership with public health authorities in 2009 in response to the H1N1 influenza pandemic, during which significant benefits and capabilities were realized ((10)).

Also, in the context of the 2009 H1N1 pandemic, Web Data showed rapid adaptability for data collection and analysis in response to the detection of the H1N1 influenza virus in swine. In this instance, CNPHI worked in partnership with the Canadian Animal Health Surveillance Network (CAHSN) of the Canadian Food Inspection Agency. Canadian Network for Public Health Intelligence and CAHSN undertook rapid adaptations and quickly commenced data collection from federal, provincial and university animal health laboratories across Canada, at a time when the impacts of H1N1 influenza (swine flu), as an animal health threat, and its interspecies transmissibility were not yet known ((11)).

Working with researchers and child welfare institutions, CNPHI also applied Web Data to assist in exploring the feasibility and benefits of abstracting added surveillance value from child welfare administrative data by coding categories of child maltreatment. Web Data questionnaires were used to analyze and assess the reliability and level of agreement with which individuals coded specific categories of child maltreatment. Results showed that coding of information from child welfare files had good potential for adding value to broader surveillance of child maltreatment to support research, policy development and decision-making ((12)).

As a means of leveraging enhanced public health surveillance and response capabilities from mobile devices and field sensors, CNPHI worked in partnership with Health Canada, First Nations and Inuit Health Branch (Alberta Region) and Sunnybrook Research Institute on a project called “CNPHI on the Go”. This initiative successfully applied Web Data technologies to enable data collection and analysis as well as two-way communication between the mobile environment and the CNPHI platform ((13)), a capability with numerous applications of benefit.

With the arrival of the COVID-19 pandemic in Canada, there was an urgent requirement to rapidly adapt the existing data collection tools used by CPSP in order to implement surveillance of emerging cases of COVID-19 among children and youth seen by paediatric practitioners.

## Intervention

The existing e-CPSP system on CNPHI, in place prior to the emergence of the COVID-19 pandemic, offered two types of data collection instruments. These included Type 1 Surveys, designed for singular monthly responses for data collection related to established studies undertaken by CPSP researchers, and Type 2 Surveys, designed for singular responses to *ad hoc* requests for information not linked to any specific studies. With the emerging COVID-19 pandemic in early 2020, CPSP had a pressing need to modify its data collection tools in order to increase both the frequency and flexibility of data collection. Building on their established working relationship, CNPHI and CPSP worked together to initiate enhancements to the CPSP’s data collection system on CNPHI, leveraging CNPHI’s agile Web Data technology.

At the outset of the intervention, very little was known about the potential impacts of COVID-19-related illness among children and youth. Given the unknown nature of the emerging public health threat, CPSP needed to focus on collecting broad information of surveillance value at a much greater frequency. Recognizing the need to commence data collection without delay, CNPHI and CPSP worked together to establish an innovative process to initiate rapid data collection in full anticipation of the need for flexibility to accommodate unknown challenges and adaptations.

Using the built-in interactive survey designer within Web Data technology, a Type 3 Survey was developed within a few days, allowing multiple responses from participants to capture weekly reporting of emerging COVID-19 cases. Type 3 Surveys were significantly longer and more complex—designed to enable the concurrent collection of more detailed information, such as demographic, epidemiological, microbiological and clinical data including comorbidities of cases identified. Despite their length and complexity, Type 3 Surveys needed to retain the flexibility to accommodate survey content enhancements to adapt to details yet unforeseen at the outset of the pandemic, such as PIMS/MIS-C.

With data collection underway, CNPHI undertook rapid enhancements to the Web Data application to accommodate the length of the Type 3 Surveys (hundreds of questions), non-responses, as well as multiple responses from individual participants. The dynamic, interactive form builder supported versatile data collection by offering a wide variety of response fields ranging from pick lists, date fields, free text and drop-down menus, for example. This versatility was fundamentally important to the collection of required information of surveillance value, yielding data fields to support queries and analysis of survey responses.

Web Data also supported ongoing adaptability to accommodate adjustments arising due to changing case definitions or feedback received. With the capability to produce the surveys in English and French, adaptability also accommodated the need to adjust translated terms to maintain accuracy, consistency and clarity throughout the process.

**Figure 1:**
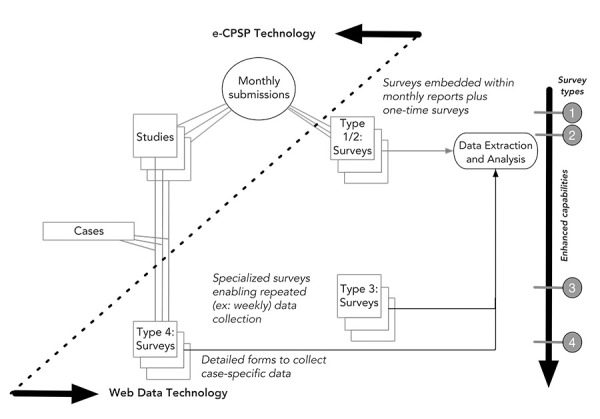
A depiction of the enhancements to the Canadian Paediatric Surveillance Program’s web-based system on CNPHI in response to COVID-19 Abbreviation: CPSP, Canadian Paediatric Surveillance Program

Importantly, Web Data is intuitive and user-friendly. With the shared experience and lessons learned from the rapid implementation of Type 3 Surveys, CNPHI and CPSP worked towards the creation of Type 4 Surveys. Building on the expertise and confidence gained by CPSP researchers and staff, Type 4 Surveys yielded an in-house capability to design, deploy and manage data collection specific to a targeted study or cases reported. This has further increased CPSP’s preparedness and agility to adapt to emerging issues and implement rapid studies while providing a detailed data collection instrument to advance research and intelligence generation. The Type 4 Survey is currently being used by CPSP to capture data on an unintended consequence of the pandemic response: a worrisome and rapid rise in first-time hospitalizations of patients with anorexia nervosa since the arrival of COVID-19 ((14)). Below, **Table 1** provides a summary of the attributes and objectives of the four types of surveys and **Figure 1** depicts the enhancements made to the e-CPSP system on CNPHI in response to COVID-19. Type 4 Surveys now support every new study undertaken by CPSP, with all data collection taking place online.

**Table 1:** Summary of enhancements to Canadian Paediatric Surveillance Program data collection instruments in response to COVID-19

Survey type	Participant responses	Frequency	Objectives
**CPSP data collection instruments prior to COVID-19**			
Type 1	Singular	Monthly	Data collection for established research studies
Type 2	Singular	*Ad hoc*	Data collection not linked to any established research studies
**CPSP data collection instrument enhancements in response to COVID-19**			
Type 3	Multiple	Weekly	Lengthy, detailed data collection on COVID-19 cases, adaptable to changing case definitions, feedback received and changing terminology in English and French
Type 4	Per case	Per case	To establish an in-house capability and preparedness to design, deploy and manage detailed data collection per case reported within a given study

Abbreviations: COVID-19, coronavirus disease 2019; CPSP, Canadian Paediatric Surveillance Program

## Outcomes

The creation and deployment of the Type 3 surveys occurred in rapid fashion, enabling CPSP to initiate detailed COVID-19 case surveillance by April 8, 2020, less than one month after the World Health Organization declared the COVID-19 pandemic. As the pandemic unfolded, numerous adaptations and modifications to the Type 3 Surveys were successfully completed to keep pace with changing needs. By May 2020, with the arrival of PIMS/MIS-C, which was a new condition temporally associated with COVID-19, CPSP researchers were able to quickly pivot and adapt the survey tool to capture critical information on this new condition.

Importantly, the adaptations made to CPSP’s system on CNPHI have also established an ongoing capability for CPSP to quickly respond to changing surveillance needs. Study investigators now have access to the online data, which is far more comprehensive than data collected via the hard copy forms. Also, in response to cases reported, CPSP can now respond with an online questionnaire to the reporting physician either the same day or the following day, a vast improvement in timeliness compared to the mailed questionnaire that could take a week to reach the reporting physician. This overall effort became a staging ground for Type 4 Surveys, yielding the preparedness and confidence for CPSP to advance towards autonomous management of the data collection and analytical tools offered by Web Data technologies on the CNPHI platform and the readiness to apply them in the future when the need arises.

Web Data provided an agile environment to support rapid survey development as a means of detailed data collection. The interactive form builder offered sufficient data field flexibility to support the complexity and length of the surveys while maintaining an ongoing ability to accommodate changes and enhancements over time, in English and French. Data extraction and analytical capabilities within Web Data enabled optimal surveillance value to be gleaned from the data collected.

## Discussion

The long-standing CNPHI-CPSP partnership has provided an environment of familiarity that comes with a history of working together. A shared knowledge of CPSP’s existing surveillance tools and strategies allowed the partners to immediately focus on the adaptations needed to implement a timely surveillance response in the face of a novel public health threat. This, together with the ready availability of the agile Web Data technology, allowed for the successful achievement of the objective of enabling CPSP to adapt and respond in timely fashion to characterize the impacts of COVID-19 on Canadian children and youth.

Important non-technical aspects contributed to the agility of this response. Logic may dictate that when one is faced with an emerging public health threat, it is not the right time to start getting to know the role and function of your key partners. This sentiment is clearly reflected in discussions that seek to define the elements of preparedness and resilience. As a program dedicated to excellence in providing scientific public health informatics solutions across a wide array of public health disciplines, CNPHI places a fundamental importance on collaborative partnerships by placing them at the core of its philosophy. With Type 3 surveys deployed and data collection underway, the pandemic was still in its first month. To prepare for the unknown road ahead, the partners built on their experience to establish an in-house capacity for CPSP to design, deploy and manage Type 4 surveys using Web Data, enhancing their ongoing preparedness and agility to respond to the unforeseen.

## Limitations

A diminishing proportion of CPSP’s community of participants has not yet made the transition to electronic reporting through CPSP’s system on CNPHI. As a result, a paper-based system is still maintained for some participants, which requires extra time and effort for CPSP in terms of data collection, extraction and analysis.

However, as an indirect benefit, with mailing methods also impacted by pandemic-related restrictions and office closures, many remaining paper-based participants elected to make the transition to electronic reporting. There are now fewer than 100, out of approximately 2,800 participants still using the paper-based reporting system—a substantial decline compared to before the pandemic.

With electronic data collection through Web Data, quality assurance steps were required to identify and address duplicate responses in some instances. Furthermore, although CPSP has reached a comfort level for managing and deploying Type 4 Surveys using Web Data, researchers and members of the medical community will still need to achieve consensus on survey and study design with respect to emerging issues of concern. Finally, the creation of detailed surveys in more than one language introduces a need for vigilance to ensure that the accuracy and consistency of translated terms are maintained, particularly in the context of an emerging infectious disease with impacts yet unknown, as terminology and case definitions may vary.

## Conclusion

With the arrival of a novel public health threat with impacts yet unknown, CNPHI and CPSP successfully leveraged their long-standing partnership to complete rapid and complex enhancements to existing data collection instruments, enabling CPSP to successfully adapt and begin characterizing the impacts of COVID-19 on Canadian children and youth, less than a month after the World Health Organization declared the pandemic. The strategic importance of this established, long-standing partnership cannot be overstated, as a key element contributing the timeliness and agility of this response.

The Canadian Network for Public Health Intelligence’s innovative Web Data technology proved to be agile, adaptable and robust, enabling the rapid creation of lengthy and complex surveys for data collection and the subsequent extraction and analysis of data collected.

Innovative technologies and established partnerships were shown to be important components of preparedness in the face of a novel public health threat.
